# Assessing the Efficacy of an Educational Smartphone or Tablet App With Subdivided and Interactive Content to Increase Patients’ Medical Knowledge: Randomized Controlled Trial

**DOI:** 10.2196/10742

**Published:** 2018-12-21

**Authors:** Thomas Timmers, Loes Janssen, Yvette Pronk, Babette C van der Zwaard, Sander Koëter, Dirk van Oostveen, Stefan de Boer, Keetie Kremers, Sebastiaan Rutten, Dirk Das, Rutger CI van Geenen, Koen LM Koenraadt, Rob Kusters, Walter van der Weegen

**Affiliations:** 1 Interactive Studios Rosmalen Netherlands; 2 Radboud University Medical Center IQ Healthcare Nijmegen Netherlands; 3 VieCuri Medical Centre Venlo Netherlands; 4 Kliniek ViaSana Mill Netherlands; 5 Jeroen Bosch Ziekenhuis s-Hertogenbosch Netherlands; 6 Canisius-Wilhelmina Hospital Nijmegen Netherlands; 7 Sint Anna Ziekenhuis Geldrop Netherlands; 8 Amphia Hospital Breda Netherlands; 9 Open University of The Netherlands Heerlen Netherlands

**Keywords:** patient education, shared decision making, smartphone, decision aid, orthopedics

## Abstract

**Background:**

Modern health care focuses on shared decision making (SDM) because of its positive effects on patient satisfaction, therapy compliance, and outcomes. Patients’ knowledge about their illness and available treatment options, gained through medical education, is one of the key drivers for SDM. Current patient education relies heavily on medical consultation and is known to be ineffective.

**Objective:**

This study aimed to determine whether providing patients with information in a subdivided, categorized, and interactive manner via an educational app for smartphone or tablet might increase the knowledge of their illness.

**Methods:**

A surgeon-blinded randomized controlled trial was conducted with 213 patients who were referred to 1 of the 6 Dutch hospitals by their general practitioner owing to knee complaints that were indicative of knee osteoarthritis. An interactive app that, in addition to standard care, actively sends informative and pertinent content to patients about their illness on a daily basis by means of push notifications in the week before their consultation. The primary outcome was the level of perceived and actual knowledge that patients had about their knee complaints and the relevant treatment options after the intervention.

**Results:**

In total, 122 patients were enrolled in the control group and 91 in the intervention group. After the intervention, the level of actual knowledge (measured on a 0-36 scale) was 52% higher in the app group (26.4 vs 17.4, *P*<.001). Moreover, within the app group, the level of perceived knowledge (measured on a 0-25 scale) increased by 22% during the week within the app group (from 13.5 to 16.5, *P*<.001), compared with no gain in the control group.

**Conclusions:**

Actively offering patients information in a subdivided (per day), categorized (per theme), and interactive (video and quiz questions) manner significantly increases the level of perceived knowledge and demonstrates a higher level of actual knowledge, compared with standard care educational practices.

**Trial Registration:**

International Standard Randomized Controlled Trial Number ISRCTN98629372; http://www.isrctn.com/ISRCTN98629372 (Archived by WebCite at http://www.webcitation.org/73F5trZbb)

## Introduction

### Background

Shared decision making (SDM) refers to the process that involves the participation of both the physician and patient to select the best suitable treatment, taking into account the clinical data and patients’ preferences and expectations [1]. Modern health care increasingly focuses on SDM because of its positive effects on patient satisfaction, therapy compliance, and outcomes [2,3]. One of the key drivers of SDM is the patients’ knowledge about the illness and treatment options available [4]. This knowledge is typically acquired through patient education, and that knowledge is currently primarily transmitted during medical consultations. Unfortunately, previous studies have shown that patients scarcely remember doctors’ reports after their consultations and that their memory for medical information is substantially limited [5]. Indeed, recent research has indicated that, on average, approximately 40% to 80% of the information provided to patients by health care practitioners was immediately forgotten, and out of what the patients did recall, approximately half of the content was inaccurate [6].

Several factors, some of which are difficult to change, contribute to poor memory acquisition, including patient age [7-9], patient level of education [10-12], the fact that too much information is provided in too little time [13-15], and that is likely exasperated by doctors’ busy schedules and their difficult use of language and jargon [5,16,17]. On the other hand, there are numerous factors that can positively influence patient recall, for example, subdividing the delivery of information [11,13-15] and the explicit categorization of content [18]. Furthermore, the usage of questions and feedback to test (and reflect) patients’ knowledge [11,19] as well as the specific modality of information transmission contribute to patient recall of medical information. Indeed, people tend to remember 20% auditory information, 40% of read information, and up to 80% of information acquired from interactive education [20-22].

Technology for health (electronic health [eHealth] or mobile health) plays an increasingly important facilitating role in educating patients [23]. The information is always available, consistent, and complete [24]; the doctor is always welcoming; patients can determine their own pace [25]; and the information can even be tailored to patients’ personal needs [26].

**Figure 1:**
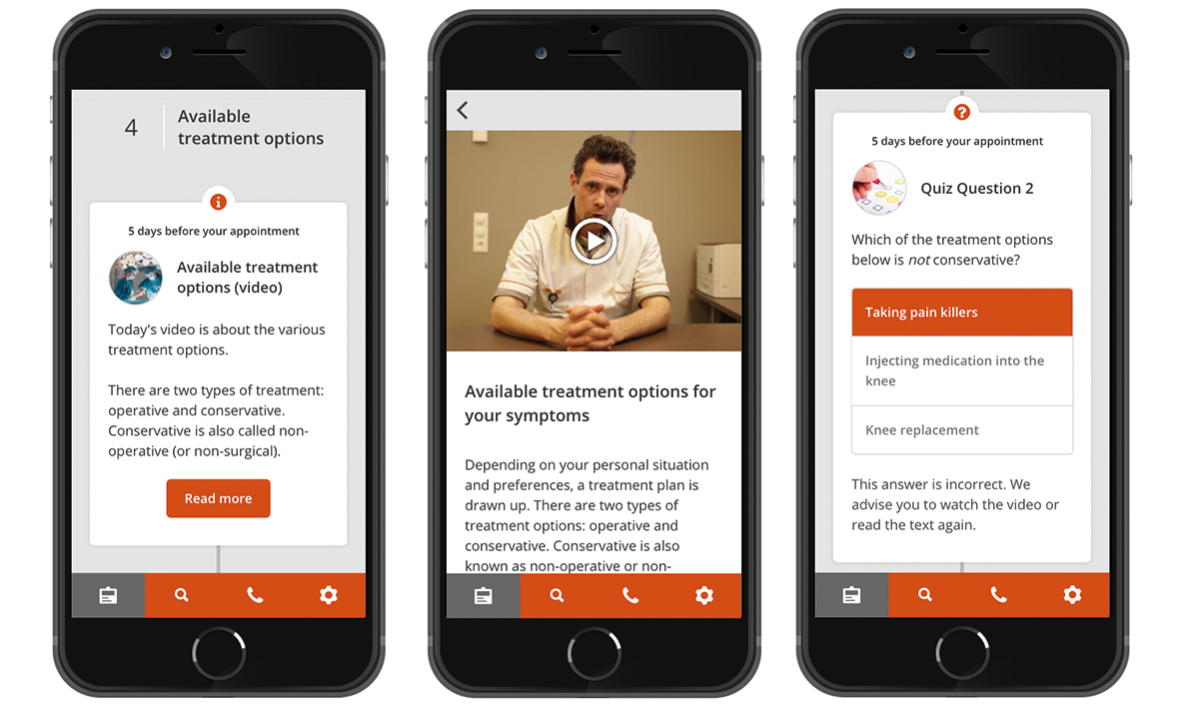
Examples of the interactive app. From left to right: the interactive timeline, information about a certain topic, and quiz-like questions with feedback.

The goal of this study was to examine the effectiveness of using an interactive app (Figure 1) in patients who were referred to the hospital by their general practitioner (GP) owing to knee complaints that indicated osteoarthritis (OA). Knee OA is a progressive condition that causes pain and functional limitations [27]. In the early phase of OA, it can be treated conservatively. End-stage OA, however, is most effectively treated with joint replacement surgery [28]. Knowledge about their condition and treatment options is important for these patients to make a well-considered decision between conservative or surgical treatment.

### Objectives

We hypothesize that compared with the above-mentioned standard practices of educating patients, the use of an interactive app would lead to a higher level of knowledge (perceived and actual) about their illness and treatment options. This is the primary outcome of our study. With regard to the secondary outcomes, we hypothesize that there will be a relative increase in reported patient satisfaction concerning their level of knowledge and the amount of information available. In addition, it is hypothesized that using the interactive app would positively influence patients’ general satisfaction with their consultation and their confidence in the treatment choice they made. All outcomes are measured by means of Web-based questionnaires.

## Methods

### Study Design

A total of 6 hospitals (4 nonacademic teaching hospitals, 1 general hospital, and 1 specialized orthopedic clinic) were selected. Between April and September 2017, patients with knee complaints due to OA were asked to participate in a surgeon-blinded randomized controlled trial. In this study, the effectiveness of an interactive app on patients’ knowledge, satisfaction, and certainty about the treatment chosen was assessed. This was compared with standard education in a parallel group design with equal allocation ratio. No changes were made to the design after the study was commenced.

### Informed Consent and Ethical Consideration

Patients were asked to consider participating in the study after scheduling an appointment at one of the recruiting hospitals. Patients who were willing received an email with all the necessary study information required for informed consent. Patients were offered at least 2 days to reflect on the information. In the case of any questions, patients were informed that they could contact the local research coordinator from each specific hospital by phone or email. Patients indicated their consent by signing an online informed consent form. Patients were also informed that their data would be kept confidential and protected. There were no indicators of substantial risk as a function of participating in this study. The study was registered at ISRCTN, with reference number ISRCTN98629372. Due to technical problems during the initial, prospective registration, the study was registered retrospectively. Registration took place after the study was completed, on May 13, 2018. The study was approved by the regional Medical Ethical Board of the Maxima Medisch Centrum (Eindhoven, The Netherlands), reference number N16.130, as well as at each of the participating sites. In addition, we attest that we have obtained appropriate permissions and paid any required fees for use of copyright-protected materials.

### Participant Selection

The eligibility of patients was assessed during their first contact with the hospital to schedule their appointment with the orthopedic surgeon. Patients had to be older than 40 years and referred by their GP because of knee complaints indicating OA. Participants were required to be fluent in Dutch and in the possession of an email address and a smartphone or tablet. At least 10 days between scheduling the appointment and the hospital visit were required, to give patients in the app group the chance to experience the intervention.

### Randomization

Patients who considered participation were controlled and randomly assigned by a computer to either a control or app group as soon as they were registered in the Web-based system by the hospital staff. Randomization was performed without block or stratification restrictions. Participants were not informed of which group they were assigned to, although both groups received an email with all the information about the study. From this email, patients who chose to participate in the study could directly give their Web-based informed consent and fill out the baseline questionnaire. Patients allocated to the app group received an additional email after completing the baseline questionnaire. This email contained download instructions for the app, a Google Play and Apple App Store download link, and the patients’ personal code. Thereafter, both groups simultaneously received the same questionnaires again on 2 separate occasions: 2 days before the arranged consultation and 1 day after the consultation. Per questionnaire, a maximum of 2 email reminders was sent.

### Intervention

In this study, the Patient Journey App (Interactive Studios, Rosmalen, The Netherlands) was used as the intervention. By using push notifications, we actively offered patients information about knee OA, (conservative and operative) treatment options, risks, rehabilitation, and expectancies in a subdivided (daily) and categorized (per theme) manner. Information was presented on an interactive timeline using text, photos, and video content (Figure 1). Interactive quiz-like questions were used to test their knowledge, providing direct feedback on the given answer.

The content for the app was compiled based on the input of 10 orthopedic surgeons from various hospitals, the Dutch option grid for knee OA [29], and information booklets from 3 participating hospitals. The 5 most important topics, as agreed upon by the surgeons, were (1) knee anatomy and the origin of the complaints, (2) different types of conservative and operative treatments, (3) risks of surgery, (4) rehabilitation after total knee replacement, and (5) expectations after total knee replacement. These topics also formed the base for the questionnaires addressing perceived and actual knowledge. Both the control group and the app group had access to standard education as offered by the hospitals, consisting of at least a website and an information event. Only the app group received the app, protected with a personal code.

Patients used the app in the 7 days before the first consultation with their orthopedic surgeon. During the first 5 days, information concerning the 5 most important topics was provided, whereas on days 6 and 7, a summary as well as practical information on how to prepare for the consultation itself were provided. Patients received daily push notifications at 10:00 am. During the study, no changes or revisions to the app took place.

### Study Outcomes

Study outcomes were measured at 3 moments in time: baseline, 2 days before consultation, and 1 day after consultation (Table 1). The baseline measurement commenced directly after patients were included in the study. Due to the timing of the intervention, the first follow-up measurement was scheduled 2 days before the consultation—enabling patients to complete the questionnaires before their hospital visit. To assure accurate recall of the consultation, the third measurement was scheduled 1 day after consultation.

All measurements were performed by using patient-reported questionnaires. Most questionnaires were developed especially for this study and can be found in Multimedia Appendix 1. All self-developed questionnaires were validated by surgeons and researchers of all participating hospitals. No additional validation was performed in a patient population.

The primary outcome measure was patient knowledge about the illness and the available treatment options. We divided knowledge into 2 concepts: perceived knowledge (ie, how much do patients think they know) and actual knowledge (ie, how much do the patients actually know). The questionnaires were based on the aforementioned 5 most important topics for the first consultation. In the perceived knowledge questionnaire, patients received 5 questions, with answers ranging from 1 (no knowledge at all) to 5 (best imaginable knowledge). The perceived knowledge questionnaire had a sum score ranging from 5 to 25. Perceived knowledge was measured at baseline and 2 days before the consultation. The actual knowledge questionnaire required patients to answer 12 questions, each ranging from 0 (incorrect answer) to 3 points (correct answer). The absolute knowledge questionnaire had a sum score range from 0 to 36. Actual knowledge was measured only once, 2 days before the consultation. The actual knowledge questionnaire was administered only once, as answering it could prime patients, which could influence their performance at future participation.

As secondary outcomes, we assessed patients’ satisfaction with the provided information, satisfaction with their level of knowledge, and their need for more information. This questionnaire was developed for this trial. Numeric Rating Scale (NRS) scores were used to measure these outcomes. Questions concerning satisfaction had a range from 0 (not satisfied at all) to 10 (very satisfied). Questions concerning the need for more information had a range from 0 (no need at all) to 10 (very much in need of). Satisfaction and the need for more information were measured at baseline and 2 days before the consultation. Furthermore, 1 day after the consultation, we measured overall satisfaction with the consultation with an NRS score from 0 (not satisfied at all) to 10 (very satisfied). We also determined the level at which patients felt they had made a decision about their treatment together with their physician, with an NRS score ranging from 0 (strongly disagree) to 10 (strongly agree). Furthermore, the following items were measured: the type of treatment chosen (conservative, operative, and I don’t know), how sure patients were about their choice (NRS, from 0 [not sure at all] to 10 [very sure]), and whether their complaints were, in the end, actually because of knee OA (yes, no, and I don’t know). All tertiary outcomes were measured 1 day after the consultation. This questionnaire was developed for this trial.

As a fourth outcome, we assessed patients’ mobile device proficiency. To measure it, the Mobile Device Proficiency Questionnaire-16 [30] was used. This questionnaire addresses 8 domains, ranging from “sending an email” and “downloading apps” to “privacy” and “update settings.” Each domain is assessed by 2 questions about completing a task, measured on a Likert scale from 1 (never tried) to 5 (very easily), resulting in a sum score ranging from 16 to 80. As a Dutch version of this questionnaire was not available, it was translated from English to Dutch by 3 researchers independently. After reaching a consensus about the Dutch translation, it was translated back into English by a certified translation agency. No major differences to the original version were identified. This measure was performed at baseline.

**Table 1:** Overview of outcomes per measurement.

Baseline	2 days before consultation	1 day after consultation
Patient characteristics	Actual knowledge	Satisfaction with consultation
Perceived knowledge	Perceived knowledge	Type of treatment chosen
Satisfaction with information	Satisfaction with information	Certainty about the choice
Satisfaction with knowledge	Satisfaction with knowledge	—^a^
Need for more information	Need for more information	—
Mobile device proficiency	—	—

^a^Not applicable.

### Sample Size and Statistical Analysis

A priori sample size calculation (alpha=.05, [1−beta]=.80) based on a reported difference of 11.7% in knowledge between orthopedic patients using a decision aid or not [31] resulted in a minimum requirement of 83 patients in each study arm. Our primary analysis was conducted using an intention-to-treat approach and therefore included all randomized patients. Normally distributed continuous variables were presented as a mean value with the SD and statistically compared between the groups using independent Student *t* tests. Non-normally distributed variables were presented as a median value with the interquartile range and statistically compared between the groups using the Mann-Whitney U tests. Categorical variables were presented as number and percentage and compared between groups using chi-square tests. Within-group differences were tested using paired Student *t* tests in the case of normally distributed data or Wilcoxon signed-rank tests in the case of nonparametric data. *P* values of ≤.05 were assumed to indicate a significant difference. A “per protocol” analysis was performed for all primary and secondary outcomes to also examine to the robustness of our main results. All data were analyzed using IBM SPSS Statistics for Macintosh, version 22.0, (Armonk, USA).

## Results

### Study Sample

**Figure 2:**
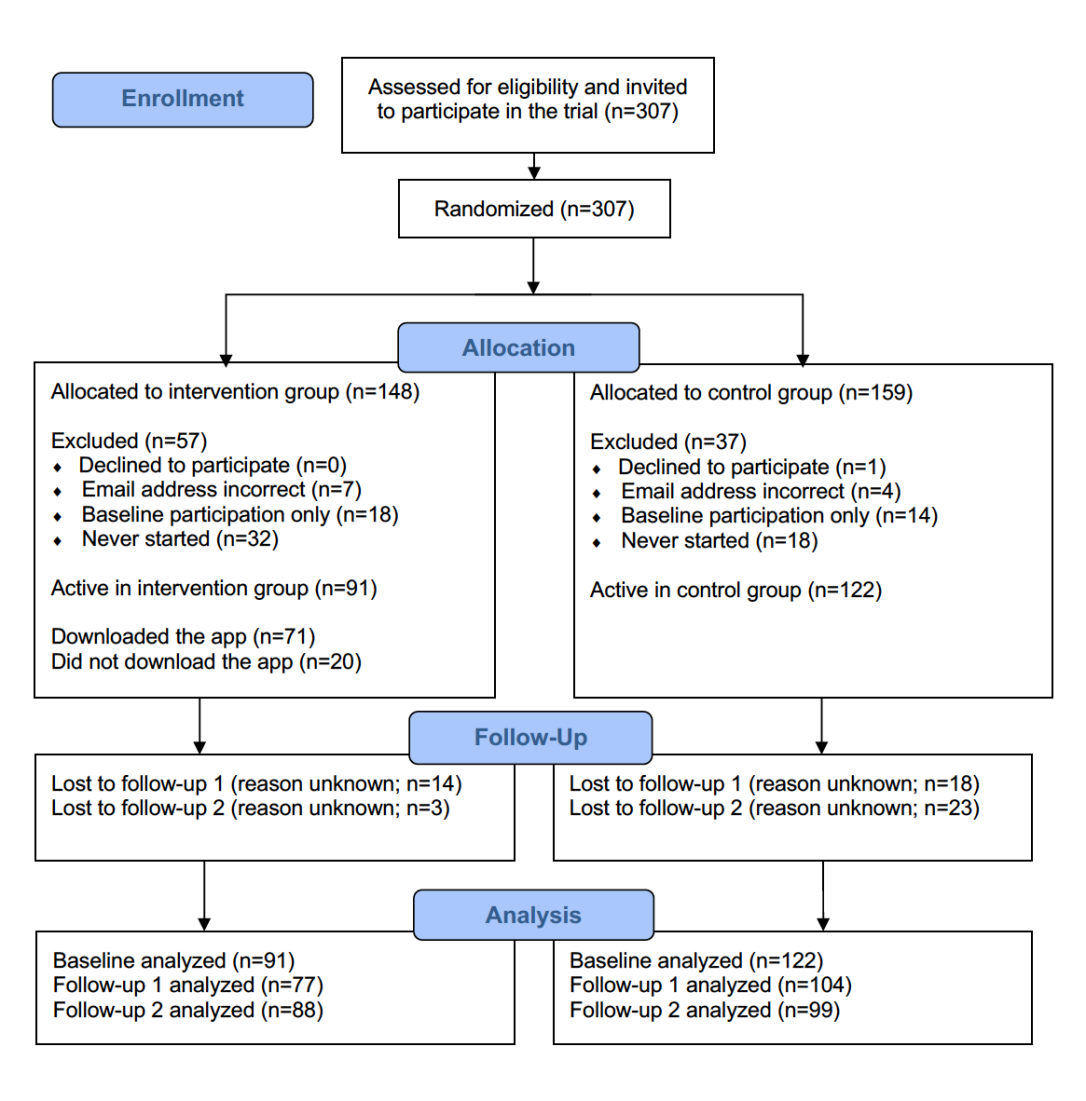
Patient flow diagram.

Between May and August 2017, a total of 307 patients considered participation in the study. A total of 50 patients (16.2%, 50/307) withdrew from the study (for reasons unknown) without completing the baseline questionnaire, and 1 patient did not consent to participate. Moreover, 11 patients (3.5%, 11/307) could not be contacted because of incorrect email addresses. In addition, 32 patients (10.4%, 32/307) participated in the baseline questionnaire but did not respond to both follow-up questionnaires (Figure 2).

Patients who missed the baseline measurement or both the follow-up questionnaires were registered as lost to follow-up. Patients whose email address was incorrectly recorded, and therefore did not receive an invitation, were registered as “email address unknown or incorrect.” Neither of them were included in the analysis. No significant differences were found between the baseline characteristics of the app group (45 male [49%], mean age=62.3 years, SD=8.3) and the control group (56 male [45%], mean age=61.8 years, SD=8.5). In addition, no significant differences were observed with respect to level of education, pain, symptoms, functional outcome, perceived knowledge, satisfaction with available knowledge, and the need for additional information (Table 2).

### Patient Knowledge Acquisition

**Figure 3:**
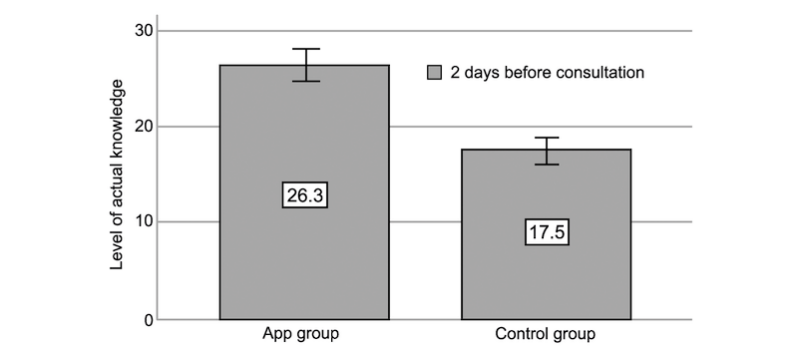
Level of actual knowledge (2 days before consultation; error bars: 95% CI).

Two days before consultation, the app group had a 52% higher level of actual knowledge (app: mean 26.4 [SD 7.4], control: mean 17.4 [SD 6.8], *P*<.001; Figure 3 and Table 3). The level of perceived knowledge was 26% higher in the app group (app: mean 16.5 [SD 3.9], control: mean 13.0 [SD 4.1], *P*<.001).

**Figure 4:**
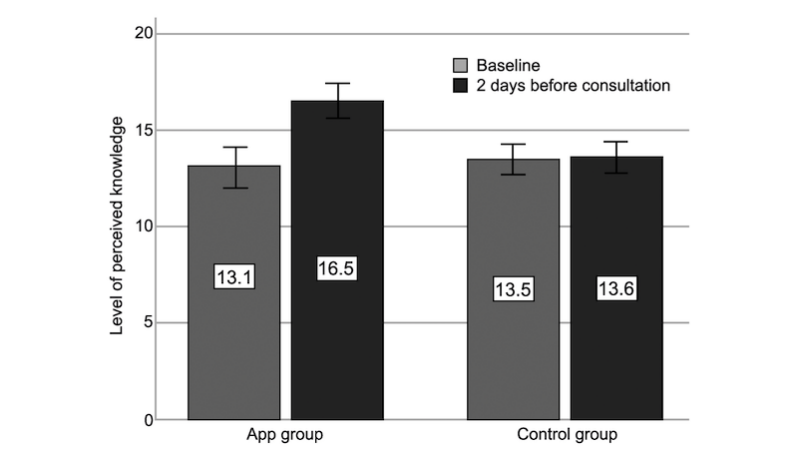
Level of perceived knowledge (baseline vs 2 days before the consultation; error bars: 95% CI).

Comparison within groups revealed an increase in perceived knowledge in the app group (baseline: mean 13.1 [SD 4.6], 2 days before consultation: mean 16.5 [SD 3.9], *P*<.001). This was not the case in the control group (baseline: mean 13.5 [SD 4.1], 2 days before consultation: mean 13.6 [SD 4.2], *P*=.78; Figure 4).

### Patient Satisfaction

Patients’ level of satisfaction with their knowledge was higher in the app group (app: mean 6.8 [SD 2.7], control: mean 5.4 [SD 2.5], *P*<.001). The level of satisfaction with the provided information was also higher in the app group (app: mean 7.0 [SD 2.3], control: mean 5.3 [SD 2.5], *P*<.001). The app group also had a lower need for additional information (app: median 7 [Q1-Q3 5-8], control: median 8 [Q1-Q3 6-9], *P*=.02).

Comparison within groups revealed an increase in patients’ satisfaction with their knowledge in the app group (baseline: mean 5.18 [SD 2.8], 2 days before consultation: mean 6.8 [SD 2.7], *P*<.001). This was not the case in the control group (baseline: mean 5.3 [SD 2.4], 2 days before consultation: mean 5.4 [SD 2.5], *P*=.57).

Comparison within groups also revealed an increase in patients’ satisfaction with the available information in the app group (baseline: mean 5.2 [SD 2.7], 2 days before consultation: mean 7.0 [SD 2.3], *P*<.001). This was not the case in the control group (baseline: mean 5.1 [SD 2.3], 2 days before consultation: mean 5.3 [SD 2.5], *P*=.56).

### Consultation

Overall satisfaction with the consultation with the orthopedic surgeon showed no difference between groups (app: median 9 [Q1-Q3 8-9], control: median 9 [Q1-Q3 7-9], *P*=.32). The extent to which patients felt they chose their treatment together with the orthopedic surgeon also did not differ between groups (app: median 9 [Q1-Q3 7-9], control: median 8 [Q1-Q3 7-8], *P*=.25).

### Treatment

Patients in the app group were more confident about their choice of treatment (app: median 8 [Q1-Q3 7-10], control: median 8 [Q1-Q3 5-8], *P*=.04). There was no difference between groups concerning the choice for conservative or operative treatment (*P*=.34). In the control group, more patients were uncertain of their choice of treatment (22.3% vs 10.1%, *P*=.03). In addition, the control group had significantly more patients that reported they had no idea whether their complaints were, in the end, caused by OA (26.3% vs 10.1%, *P*=.02).

**Table 2:** Patient characteristics.

Characteristics		App group (n=91)	Control group (n=122)	*P* value
**Sex, n (%)**				
	Male	45 (49)	56 (45.9)	—^a^
	Female	46 (50)	66 (54.1)	.61
**Education, n (%)^b^**				
	Group 1^c^	56 (62)	73 (64.0)	—
	Group 2^d^	33 (37)	41 (36.0)	.87
Duration of complaints >6 months^e,f^, n (%)		57 (62)	70 (57.4)	.44
Walking <30 min^f,g^, n (%)		75 (82)	100 (82.0)	.93
Pain during the night^f,h^, n (%)		21 (23)	28 (23.0)	.98
Age, mean (SD)		62.27 (8.32)	61.75 (8.54)	.66
KOOS PS^i,j^, mean (SD)		22.96 (5.19)	22.73 (5.44)	.75
Perceived knowledge, mean (SD)		13.04 (4.41)	13.39 (4.14)	.56
Satisfaction with knowledge, mean (SD)		5.34 (2.77)	5.22 (2.46)	.72
**Pain, mean (SD)**				
	At rest^j,k^	4.91 (2.61)	5.02 (2.58)	.77
	During activity^j,k^	7.16 (1.91)	6.94 (2.23)	.45
Mobile Device Proficiency Questionnaire-16, mean (SD)		59.31 (19.73)	60.28 (18.77)	.97
Satisfaction with information, median (Q1-Q3)		6.00 (3-7)	5.00 (4-7)	.40
Need for more information, median (Q1-Q3)		9.00 (8-10)	8.00 (7-10)	.88

^a^Not applicable.

^b^Level of education has been split into 2 groups for the purpose of analysis.

^c^Educational levels in group 1: none, elementary school, and secondary (vocational) education.

^d^Educational levels in group 2: higher secondary education, pre-university education, and university (of applied science).

^e^Duration of complaints has been split into 2 groups for analysis purposes and was measured categorically (<3 months, 3-6 months, 6-12 months, and >12 months).

^f^Typical complaints for knee osteoarthritis patients, advised by participating orthopedic surgeons.

^g^Ability to walk for 30 min was measured as a dichotomous variable. Data represents patients who answered “no”.

^h^Pain at night was measured as a dichotomous variable. Data represents patients who answered “yes”.

^i^The Knee injury and Osteoarthritis Outcome Score-Physical Function Shortform (KOOS PS) [32] was used to assess functional outcome.

^j^Part of The Netherlands Orthopaedic Association guideline for knee osteoarthritis.

^k^Pain at rest and during activity was measured using a Numeric Rating Scale (NRS) from 0 (no pain) to 10 (severe pain).

**Table 3:** Actual knowledge questionnaire and distribution of the correct answers.

Question	App, n (%)	Control, n (%)	*P* value
1. What is knee osteoarthritis? a. A type of pain relief; *b. Wear and tear of the knee joint*^a^; c. The conservative treatment of knee problems; d. I don’t know	75 (96.2)	101 (96.2)	.99
2. In what way does osteoarthritis cause knee problems? a. Poor circulation in the leg; *b. Deterioration of the cartilage quality*; c. Injury to the knee caused by work or sport; d. I don’t know	70 (89.7)	87 (82.9)	.19
3. Which of the following treatments is not a conservative treatment? a. Walking with crutches or a stick, possibly combined with physiotherapy; b. Injection in the knee; *c. Placement of a knee prosthesis*; d. I don’t know	51 (65.4)	37 (35.2)	<.001
4. What is the average life of a knee prosthesis? a. An average of 5-10 years; b. An average of 10-15 years; *c. An average of 15-20 years*; d. I don’t know	44 (56.4)	32 (30.5)	<.001
5. Which of the following preparations are important to reduce the risk of complications during an operation? More than one answer can be correct^b^; *a. Stop smoking*; *b. Certain physiotherapeutic exercises*>; c. Stop exercising; *d. Healthy eating*; e. Stop working; f. I don’t know	43 (55.1)	31 (29.5)	<.001
6. How often does the knee prosthesis become infected so that it needs to be replaced? *a. In about 1 percent of cases*; b. In about 5 percent of cases; c. In about 10 percent of cases; d. I don’t know	32 (41.0)	14 (13.3)	<.001
7. A possible complication of a knee prosthesis is thrombosis (blood clot) in the legs. How can this be avoided? a. Avoid overextending the operated leg for 4-6 weeks after the operation; b. Walk with crutches or a stick for 4-6 weeks after the operation; *c. Use blood thinners for 4-6 weeks after the operation*; d. I don’t know	58 (74.4)	50 (47.6)	<.001
8. What is the duration of the average hospital stay for patients who have received a knee prosthesis? a. *1-3 days*; b. 4-7 days; c. 7-10 days; d. I don’t know	67 (85.9)	63 (60.0)	<.001
9. How many months on average will you receive physiotherapy after you have had a knee prosthesis? a. Less than a month; b. 1-3 months; *c. 3-6 months*; d. I don’t know	41 (56.6)	29 (27.6)	<.001
10. How long on average will it take until you have fully recovered after a knee prosthesis operation? a. 1-3 months; b. 3-6 months; *c. 6-12 months*; d. I don’t know	53 (67.9)	36 (34.3)	<.001
11. Which of the following statements about a knee prosthesis are true? More than one answer can be correct^b^; *a. For many patients the pain will decrease, allowing them to move more easily*; b. It is safe to partake in activities such as basketball, football, and volley ball; *c. After 2-3 months, many patients are able to resume part of their daily activities*; *d. It is safe to partake in activities such as walking, swimming, and cycling*; e. I don’t know	55 (71.4)	54 (51.4)	.01
12. What percentage of patients will be completely without pain after receiving a knee prosthesis? a. 65-70%; b. 75-80%; *c. 85-90%*; d. I don’t know	51 (65.4)	17 (16.2)	<.001

^a^Italics indicate the correct answer.

^b^Only the combination of all 3 correct answers was indicated as “correct”.

**Table 4:** Per protocol analysis of the main outcomes (2 days before consultation).

Outcome	Intention to treat			Per protocol		
	App (n=91), mean (SD)	Control (n=122), mean (SD)	*P* value	App (n=71), mean (SD)	Control (n=142), mean (SD)	*P* value
Actual knowledge	26.4 (7.4)	17.4 (6.8)	<.001	27.6 (7.3)	17.9 (6.8)	<.001
Perceived knowledge	16.5 (3.9)	13.0 (4.1)	<.001	17.0 (3.7)	13.6 (4.1)	<.001
Satisfaction with knowledge	6.8 (2.7)	5.4 (2.5)	<.001	6.8 (2.2)	5.5 (2.5)	<.001
Satisfaction with information	7.0 (2.3)	5.3 (2.5)	<.001	7.1 (3.9)	5.4 (4.1)	<.001

### Mobile Device Proficiency of the Population

There was no difference in mobile device proficiency between groups at baseline (app: mean 59.3 [SD 19.73], control: mean 60.3 [SD 18.77]). The items most frequently referred to as “never tried” were the transferring of data to and from a mobile device, scheduling appointments in their agenda, playing games, and listening to music. All the items necessary for the use of the educational app were rated “easily” or “very easily” by >75% of patients. These items included using the device to find and start the app and using the keyboard. About 24% of the participants had never tried to search for an app in the App or Play Store.

### Per Protocol Analysis

All presented results so far were analyzed using the intention-to-treat method. Analysis based on the per protocol method also resulted in the main outcomes being in favor of the app group, albeit somewhat more pronounced (Table 4).

## Discussion

### Principal Findings

In this study, we primarily investigated the possible effects of actively sending subdivided, categorized, and interactive information through an app on patients’ knowledge of knee complaints and their treatment options. In addition, patient satisfaction with provided information, knowledge, and the consultation as well as patient self-reported confidence in treatment choice were measured. In comparison with patients who received standard care, the level of actual knowledge was 52% higher in patients who used the app designed for this study. This approach seems to be much more effective compared with the use of decision aids as described in the Cochrane 2017 systematic review, in which a total of 52 studies, calculating for 13,316 patients, were included, and knowledge increased by 13.27% [3].

Apart from actual knowledge, patients in the app group also experienced a significantly higher level of perceived knowledge about their illness and treatment options, whereas in the control group, there was hardly any change. The app group rated their level of perceived knowledge as 16.5 out of 20 (ie, 8.3 on a 0-10 scale). The control group rated their level of knowledge as 13.5 out of 20 (ie, 6.8 on a 0-10 scale). Their level of actual knowledge, however, demonstrated that they sometimes had little to very little knowledge about the topics (see Table 3). This *overestimation* of one’s knowledge fosters their confidence of being capable of choosing the right treatment [33]. Choosing a treatment based on nonexistent or wrong information, however, is a known predictor for dissatisfaction [34-36].

Patients in the app group not only had more knowledge but were also more satisfied with their knowledge and the information they received and were to a lesser extent in need of more information. This is in line with earlier research, describing increased satisfaction when one is offered a decision aid [3]. Nonetheless, no differences were found between the groups in the way they experienced their consultation with their physician nor in the extent to which they felt they had made the choice for their treatment together with their physician.

On the basis of these results, one might question patients’ need for knowledge. However, previous studies have demonstrated that when patients are more knowledgeable, they have less decisional conflict [3]. This is arguably supported by our finding that patients in the app group were more confident about their chosen treatment. Furthermore, in the days directly following the consultation, 90.2% of patients in the app group could remember the type of treatment chosen versus 78.3% in the control group (*P*=.02). In addition, the number of patients who did not know whether their complaints were caused by OA was smaller in the app group (10.3%) than in the control group (26.7%; *P*=.01).

### Comparison With Prior Work

To try to explain the large difference in actual knowledge gain, we took a closer look at the Cochrane 2017 review. This review updated the Cochrane 2014 review on the use of decision aids, to which 18 new studies were added. We examined these 18 studies, assuming they would provide an up-to-date overview of the types of interventions used in recent years. All newly added studies were conducted between 2012 and 2015, except for one that was conducted in 2006. In these studies, booklets, DVDs, websites, one-on-one conversations, phone calls, and group sessions were used as decision aids. Decision aids were made available during consultations, between consultations, or after consultations with doctors. Decision aids ranged from 1-page instructions to 2-hour information sessions online or on-site.

In contrast to these studies, we used an app for smartphone or tablet as an information carrier in our study. One of the characteristics of these devices, especially smartphones, is that people often carry the device with them, lowering the barrier to use them. Within the app, we used a combination of known mechanisms on information retention: small bits of information [11,13-15], information about specific themes [6], multiple modes of information [37], and quiz-like questions with instant feedback to test (and reflect) patients’ knowledge [11,19]. None of the studies in the Cochrane review used this combination of mechanisms. Type of information carrier was often the limiting factor for using different mechanisms, as you cannot, for instance, offer subdivided content on a piece of paper, small bits of information in a 2-hour group session, or multiple modes of information in a phone call.

Another important and distinctive factor that we believe contributed to the higher level of knowledge was the usage of (daily) push notifications—actively bringing information to patients, reminding them about the information in the app, and giving them the opportunity to directly access the information by clicking on the notification. In our study, the median number of times patients viewed the information in the week before the consultation was 25 (Q1-Q3: 12-39). Sending push notifications had a direct effect on usage of the app in terms of patients opening the app, viewing information, watching a video, or answering a quiz-like question.

### Strengths and Limitations

To our knowledge, this is the first study that investigated patient education through an interactive app, while taking actual and perceived knowledge as well as satisfaction and confidence about treatment choice into account. Second, the study covers a relevant topic in modern health care: eHealth. The third strength is the design of the trial: multicenter (6 hospitals), randomized, controlled, and blinded for the treating physician. Finally, the content for the app was composed by using multiple sources of information, including orthopedic surgeons and current guidelines.

A limitation of our study is the fact that the level of actual knowledge was only measured once. Therefore, we could not perform a within-group comparison to assign the higher level of actual knowledge to the intervention. However, the randomization, the similarity of baseline characteristics between groups, and the significant increase in perceived knowledge only in the intervention group render it likely that the difference in actual knowledge between groups is because of the intervention.

Second, 22% of patients in the app group did not download the app, for unknown reasons. Perhaps, the instructions were too complex or the patients had trouble with the initial download from the App or Play Store. This demonstrates the necessity to offer support to patients for the initial usage of interventions such as these. Nevertheless, the level of adherence was high (70%). Moreover, even without correcting for this, our results show a clear advantage in the level of knowledge in the app group. This effect of the app became more pronounced when data were analyzed using the *per protocol* method. Third, we only included patients in possession of an email address and a smartphone or tablet. These criteria could limit the generalizability of the results. However, majority of the sample did use email (45-65 years: 92.9%; >65 years: 62.2%) and have a smart device (45-65 years: 92.4%; >65 years: 68.7%) [38], and this number will most probably only increase in the future. The fourth limitation is the use of self-created questionnaires to measure perceived and actual knowledge, as validated questionnaires covering our *top 5* important topics do not exist. To minimize this limitation, these questionnaires were developed in close cooperation with practicing clinicians and in line with Dutch guidelines and patient educational booklets. Finally, we did not consider individual preferences of patients regarding education on treatment options, nor their desire, or the lack of it, to participate in SDM. Earlier research has shown that some older people feel that “the doctor knows best” and that “their own knowledge is superfluous in the decision-making process” [4].

### Conclusions

Modern health care is more and more focused on patient involvement, in which knowledge about ones’ illness and treatment options is a must. Our results show that educating patients by using an interactive app with timely information could play an important role in increasing knowledge—one of the key drivers for SDM. It could benefit the consultation with the physician as well as result in long-term satisfaction with the treatment chosen.This study focused on patients being referred to an orthopedic surgeon by their GP with complaints indicating knee OA. We hypothesize, however, that neither the illness or the (phase of the) treatment limits the extent to which this type of intervention could be useful in improving patient education. Future research is needed to show the generalizability of using an app to actively offer patients subdivided and interactive information related to their specific illness or treatment. Furthermore, the (long-term) effects of this intervention on the final choice patients made regarding their treatment and the effect to which they were satisfied with this choice need to be demonstrated.

We found that, in comparison with standard educational tools, using an app to actively educate patients with subdivided, categorized, and interactive content significantly increased their level of perceived knowledge. Furthermore, a significantly higher level of actual knowledge was demonstrated in the intervention group. Patients in the app group were also more satisfied with the information they received and with their level of knowledge compared with the control group. Even though the intervention did not have an effect on their appreciation of the consultation with the doctor, patients in the app group were more confident and aware about their choice of treatment.

## Appendix

Multimedia Appendix 1 Overview of the self-developed questionnaires used in this study.

Multimedia Appendix 2 CONSORT‐EHEALTH checklist (V 1.6.1).

## Abbreviations

eHealth: electronic health
GP: general practitioner
NRS: Numeric Rating Scale
OA: osteoarthritis
SDM: shared decision making
