# Supplementary material for: Assessing the Efficacy of an Educational Smartphone or Tablet App With Subdivided and Interactive Content to Increase Patients’ Medical Knowledge: Randomized Controlled Trial
Source: JMIR Mhealth Uhealth. 2018 Dec 21;6(12):e10742. doi: 10.2196/10742 (PMC6320423; doi:10.2196/10742)
Supplement: Multimedia Appendix 1 [file mhealth_v6i12e10742_app1.pdf]

Questionnaire 1: Actual knowledge

1. What is knee osteoarthritis?<br><br>a. A type of pain relief<br>b. Wear and tear of the knee joint *<br>c. The conservative treatment of knee problems<br>d. I don’t know
2. In what way does osteoarthritis cause knee problems?<br><br>a. Poor circulation in the leg<br>b. Deterioration of the cartilage quality *<br>c. Injury to the knee caused by work or sport<br>d. I don’t know
3. Which of the following treatments is not a conservative treatment?<br><br>a. Walking with crutches or a stick, possibly combined with physiotherapy<br>b. Injection in the knee<br>c. Placement of a knee prosthesis *<br>d. I don’t know
4. What is the average life of a knee prosthesis?<br><br>a. An average of 5 to 10 years<br>b. An average of 10 to 15 years<br>c. An average of 15 to 20 years *<br>d. I don’t know
5. Which of the following preparations are important to reduce the risk of complications during an operation? More than one answer can be correct. **<br><br>a. Stop smoking *<br>b. Certain physiotherapeutic exercises *<br>c. Stop exercising<br>d. Healthy eating *<br>e. Stop working<br>f. I don’t know
6. How often does the knee prosthesis become infected so that it needs to be replaced?<br><br>a. In about 1 percent of cases *<br>b. In about 5 percent of cases<br>c. In about 10 percent of cases<br>d. I don’t know
7. A possible complication of a knee prosthesis is thrombosis (blood clot) in the legs. How can this be avoided?<br><br>a. Avoid overextending the operated leg for 4 to 6 weeks after the operation<br>b. Walk with crutches or a stick for 4 to 6 weeks after the operation<br>c. Use blood thinners for 4 to 6 weeks after the operation *

d. I don’t know
8. What is the duration of the average hospital stay for patients who have received a knee prosthesis?<br><br>a. 1 to 3 days *<br>b. 4 to 7 days<br>c. 7 to 10 days<br>d. I don’t know
9. How many months on average will you receive physiotherapy after you have had a knee prosthesis?<br><br>a. Less than a month<br>b. 1 to 3 months<br>c. 3 to 6 months *<br>d. I don’t know
10. How long on average will it take until you have fully recovered after a knee prosthesis operation?<br><br>a. 1 to 3 months<br>b. 3 to 6 months<br>c. 6 to 12 months *<br>d. I don’t know
11. Which of the following statements about a knee prosthesis are true? More than one answer can be correct. **<br><br>a. For many patients the pain will decrease, allowing them to move more easily*<br>b. It is safe to partake in activities such as basketball, football and volley ball<br>c. After 2 to 3 months, many patients are able to resume part of their daily activities*<br>d. It is safe to partake in activities such as walking, swimming and cycling*<br>e. I don’t know
12. What percentage of patients will be completely without pain after receiving a knee prosthesis?<br><br>a. 65 to 70 percent<br>b. 75 to 80 percent<br>c. 85 to 90 percent *<br>d. I don’t know

* indicates the correct answer
** only the combination of all 3 correct answers was indicated as “correct”

Questionnaire 2: Perceived knowledge questionnaire

How much knowledge do you currently have about the following subjects:

	Very much knowledge	Much knowledge	Neutral	Little knowledge	Very little knowledge
1. The functioning of your knee and the origin of your complaints					
2. Available treatments for wear and tear of the knee					
3. Risks and possible complications of surgery					
4. Rehabilitation program (activities and duration)					
5. What you can expect from a knee prosthesis					

Questionnaire 3: Knowledge & Satisfaction

1. How satisfied are you with your current knowledge about your knee problems and the possibilities to treat them?

Not satisfied at all 0 1 2 3 4 5 6 7 8 9 10 Very much satisfied

2. How satisfied are you with the information you have received so far about your knee problems and the possibilities to treat them?

Not satisfied at all 0 1 2 3 4 5 6 7 8 9 10 Very much satisfied

3. Do you need more information about your knee problems and the possibilities to treat them?

No need at all 0 1 2 3 4 5 6 7 8 9 10 Very much need

Questionnaire 4: Consultation with your doctor

1. How satisfied are you, in general, about the consultation with your doctor?

Not satisfied at all 0 1 2 3 4 5 6 7 8 9 10 Very much satisfied

2. Do you feel that you were able to make a decision about the treatment of your knee problems together with your doctor?

Not at all 0 1 2 3 4 5 6 7 8 9 10 Very much indeed

3. Which treatment do you currently consider for your knee problems?

a) Lifestyle changes
b) Physiotherapy
c) Injections / Pain medication
d) Knee replacement surgery
e) Osteotomy (shinbone of thighbone correction)
f) Brace
g) Other
h) I don’t know

4. How certain are you about your choice for this treatment?

Not sure at all 0 1 2 3 4 5 6 7 8 9 10 Very sure

5. Are your knee complaints caused by wear and tear (osteoarthritis) of the knee?

a) Yes
b) No
c) I don’t know
